# In Vivo Analysis of the Biocompatibility and Macrophage Response of a Non-Resorbable PTFE Membrane for Guided Bone Regeneration

**DOI:** 10.3390/ijms19102952

**Published:** 2018-09-27

**Authors:** Tadas Korzinskas, Ole Jung, Ralf Smeets, Sanja Stojanovic, Stevo Najman, Kristina Glenske, Michael Hahn, Sabine Wenisch, Reinhard Schnettler, Mike Barbeck

**Affiliations:** 1 Section for Regenerative Orofacial Medicine, Department of Oral and Maxillofacial Surgery, University Hospital Hamburg-Eppendorf, 20246 Hamburg, Germany; tadaskorzinskas@yahoo.de (T.K.); ol.jung@uke.de (O.J.); r.smeets@uke.de (R.S.); 2 Institute of Biology and Human Genetics, Department for Cell and Tissue Engineering, University of Niš, Faculty of Medicine, 18106 Niš, Serbia; s.sanja88@gmail.com (S.S.); stevo.najman@gmail.com (S.N.); 3 Clinic of Small Animals, c/o Institute of Veterinary Anatomy, Histology and Embryology, Justus Liebig University of Giessen, 35390 Giessen, Germany; Kristina.Glenske@vetmed.uni-giessen.de (K.G.); Sabine.Wenisch@vetmed.uni-giessen.de (S.W.); 4 Department of Osteology and Biomechanics, University Hospital Hamburg-Eppendorf, 20246 Hamburg, Germany; hahn@uke.de; 5 University Medical Center, Justus Liebig University of Giessen, 35390 Giessen, Germany; reiner.schnettler@mac.com; 6 BerlinAnalytix GmbH, 12109 Berlin, Germany

**Keywords:** PTFE membrane, collagen membrane, biocompatibility, tissue reaction, inflammation, macrophage, M1, M2

## Abstract

The use of non-resorbable polytetrafluoroethylene (PTFE) membranes is indicated for the treatment of large, non-self-containing bone defects, or multi-walled defects in the case of vertical augmentations. However, less is known about the molecular basis of the foreign body response to PTFE membranes. In the present study, the inflammatory tissue responses to a novel high-density PTFE (dPTFE) barrier membrane have preclinically been evaluated using the subcutaneous implantation model in BALB/c mice by means of histopathological and histomorphometrical analysis methods and immunohistochemical detection of M1- and M2-macrophages. A collagen membrane was used as the control material. The results of the present study demonstrate that the tissue response to the dPTFE membrane involves inflammatory macrophages, but comparable cell numbers were also detected in the implant beds of the control collagen membrane, which is known to be biocompatible. Although these data indicate that the analyzed dPTFE membrane is not fully bioinert, but its biocompatibility is comparable to collagen-based membranes. Based on its optimal biocompatibility, the novel dPTFE barrier membrane may optimally support bone healing within the context of guided bone regeneration (GBR).

## 1. Introduction

Guided bone regeneration (GBR) is widely used in the fields of periodontology, implant dentistry, and maxillofacial surgery. Dental barrier membranes allow for the formation and maintainance of spaces, which, when filled with bone substitutes, stabilizes blood clots and allow the migration of osteoprogenitor cells in to the space intended for bone regeneration, while preventing the area from soft tissue penetration or collapse [1,2]. In this context, barrier membranes have to fulfill the following main criteria: separation of hard and soft tissue up to the time point of completed bone regeneration, biocompatibility, space-maintenance, cell-occlusiveness, tissue integration, and clinical manageability, amongst other different requirements [3]. Different biological and physical properties of the variety of available barrier membranes contribute to clinical decision making regarding their indications for use.

Resorbable barrier membranes are based on natural or synthetic resorbable polymers, and they are widely used for GBR [4]. Most of the resorbable barrier membranes are based on collagen, which is derived from different sources, i.e., different species such as pigs or cattle, and different harvesting sites such as the subcutaneous connective tissue or the pericardium [5]. Altogether, collagen-based barrier membranes have most often been shown to be very biocompatible biomaterials, and they allow for a comparable degree of bone regeneration, like non-resorbable membranes [6,7]. Collagen membranes avoid the need for second-stage surgery for removal, they are easy to handle, are cost effective, and show less morbidity [8]. However, the disadvantage of the collagen membranes is their lack of ability to maintain spatial stability, which in some cases leads to collapse and therefore diminished grafted bone volume [9]. Resorbable membranes of synthetic origin have also been shown to be suitable for bone regeneration. Polylactic acid (PLA)- or polylactic-co-glycolic-acid (PLGA)-based membranes provide good spatial stability of the graft material [9]. Nonetheless, these biomaterials are degraded by non-enzymatic hydrolysis and cellular metabolization under the release of acidic molecules, which negatively influences their biocompatibility and the healing process [10]. Altogether, most of the currently available resorbable barrier membranes are limited with regard to the treatment of large, non-contained bone defects or multi-walled defects, or in case of vertical augmentations.

Although second-stage surgery is required for their removal, the use of non-resorbable membranes is still indicated for GBR procedures in the case of the afore-mentioned clinical situations, as they offer a higher form of stability and space-maintaining properties [11]. Commercially available non-resorbable barrier membranes are most often made of polytetrafluoroethylene (PTFE). PTFE has been shown to be biocompatible, and it maintain its integrity during and after implantation. Some PTFE membranes are even combined with structural elements such as titanium [9]. In this context, both high density PTFE (dPTFE) and semipermeable expanded PTFE (ePTFE) membranes are available, both providing different advantages [12]. While semipermeable PTFE membranes may support a transmembraneous transport of nutrients, dense PTFE membranes have shown to act as an efficient barrier against bacterial and cellular penetration in different clinical indications, due to its small pore size [13].

Interestingly, PTFE-based biomaterials are stated to be bioinert, which means they do not induce a tissue reaction when introduced to biological tissue [14]. However, it has been shown that nearly every biomaterial induces an inflammatory tissue reaction, which is unique for every material depending on its combination of physical and chemical properties [15]. This tissue reaction to a biomaterial is a cascade including mainly macrophages as key elements, which have been shown to express both pro- and anti-inflammatory molecules depending on material factors such as surface topography or surface chemistry [16,17,18]. Based on their molecule expression, macrophages are more or less divided into pro-inflammatory M1- and anti-inflammatory M2 subtypes [19,20]. Taken together, it is believed that the successful clinical application of a biomaterial has to be accompanied by an “overall M2 tissue reaction” to promote tissue healing, while a chronic pro-inflammatory tissue response may lead to negative consequences for tissue remodeling, such as fibrous encapsulation [19,20]. Thus, the understanding of the material-specific foreign body reaction, and of the interactions of the immune system with a biomaterial is pivotal to ensure the safety, biocompatibility, and functionality of a medical device.

Interestingly, there are very limited data about the degree of the foreign body response to non-resorbable PTFE membranes. Thus, the present preclinical in vivo study aims to analyze the tissue responses to a new synthetic, non-resorbable high-density PTFE barrier membrane. Following implantation into the subcutaneous connective tissue of BALB/c mice for up to 30 days, the hypothesis of the bioinertness of PTFE-based biomaterials has been evaluated. A commercially available collagenous barrier membrane that has already been examined in different preclinical and clinical studies and described as biocompatible biomaterial was used as control material [21,22,23]. Established histopathological and histomorphometrical analysis methods, and especially immunohistochemical detection of M1- and M2-macrophages have been applied [24,25,26,27,28].

## 2. Results

### 2.1. Histological (Qualitative) Analysis

**Figure 1:**
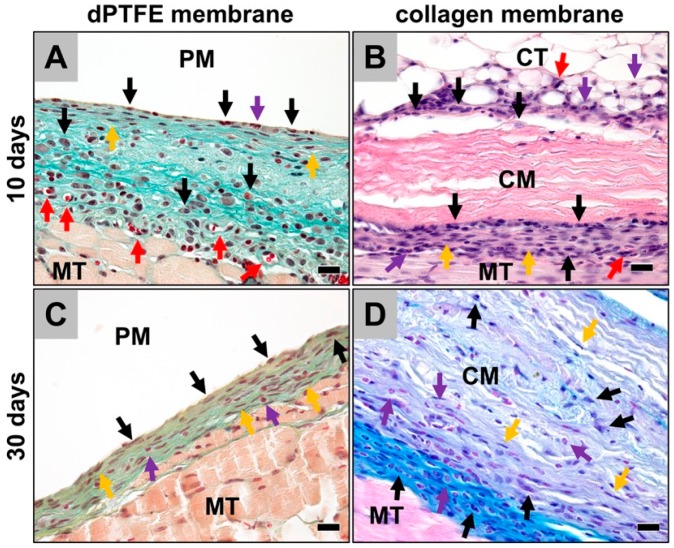
The histological images from the implantation beds of the analyzed membranes, i.e., the dPTFE membrane (PM) and the collagen membrane (CM) within the subcutaneous connective tissue (CT) (MT = muscle tissue). (**A**) At the surfaces of the dPTFE membrane, a thin layer of mononuclear cells that mainly belonged to the monocyte/macrophage line (black arrows) beside single granulocytes (purple arrow) were observable. Within the reactive peri-implant tissue, mainly macrophages (black arrows) and fibroblasts (yellow arrows) were found, besides small numbers of granulocytes and lymphocytes as well as some small vessels (red arrows) (Movat’s Pentachrome-staining, 400× magnification, scale bar = 20 µm). (**B**) Into the implant beds of the collagen membranes, a comparable tissue reaction, including mainly macrophages (black arrows) besides single eosinophils and fibroblasts (purple/yellow arrows) (haematoxylin and eosin (HE)-staining, 400x magnification, scale bar = 20 µm). At this time point only some single cells have migrated into the outer regions of the membrane body. (**C**) At day 30 after implantation, the wall of reactive tissue around the dPTFE membranes have visibly been decreased. Macrophages (black arrows) were still observed dominating the tissue reaction beside single eosinophils and fibroblasts (purple/yellow arrows) (Movat’s Pentachrome-staining, 400× magnification, scale bar = 20 µm). (**D**) Also, into the implant beds of the collagen membrane mainly macrophages (black arrows) have been detected at this time point, together with low numbers of eosinophils and fibroblasts (purple/yellow arrows). At day 30 after implantation, more cells have invaded the membranes body, while the material showed no signs of breakdown (Alcian blue-staining, 400× magnification, scale bar = 20 µm).

The results of the histological analysis showed an inflammatory tissue reaction within the implantation beds of the dPTFE membranes at day 10 post-implantation (Figure 1A). A thin reactive tissue wall was detectable surrounding the membranes, which was mainly composed of inflammatory cell types such as macrophages and granulocytes, besides single other cell types such as fibroblasts (Figure 1A). Furthermore, single vessels have been found within the reactive connective tissue (Figure 1A). At this time point, no biomaterial-associated multinucleated giant cells (BMGCs) have been observed. Furthermore, no tissue ingrowth into the membrane has been detected.

In case of the collagen membrane (control), a comparable tissue reaction has been observed (Figure 1B). Mainly cell types such as macrophages, eosinophilic granulocytes, and single fibroblasts were found within the thin walls of the reactive tissue adherent to the biomaterial (Figure 1B). Only some single cells penetrated the membrane body at this early post-implantation time point of 10 days outgoing (Figure 1B). Furthermore, no BMGCs were detected at this time point.

**Figure 2:**
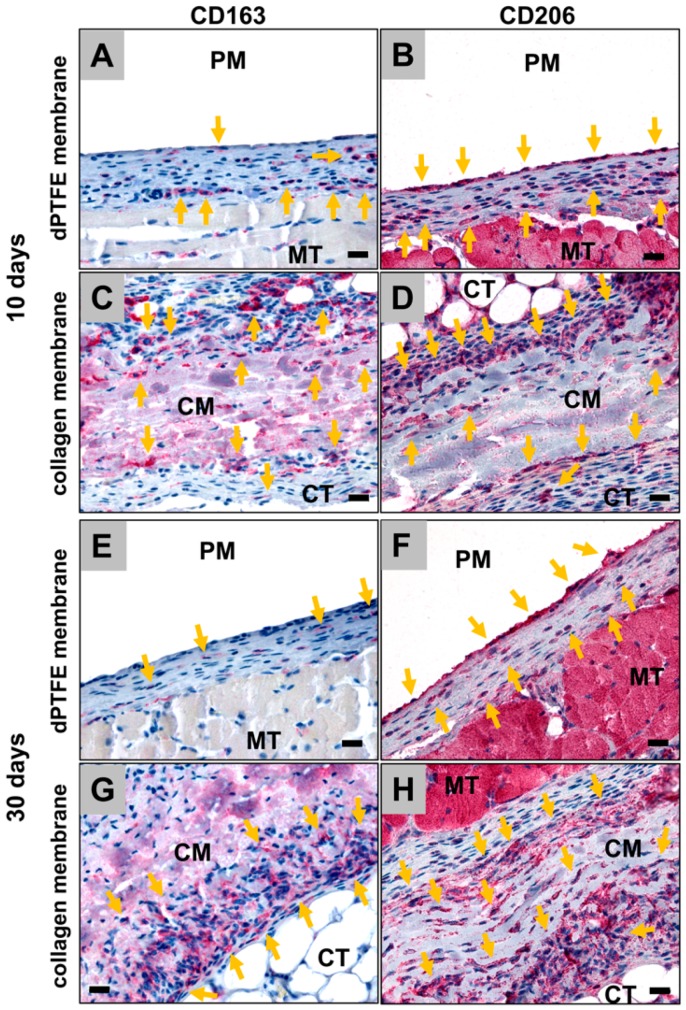
Exemplary images of the detection of M2 (CD163) and M1 (CD206) positive macrophages (yellow arrows) into the implantation beds of the dPTFE and the collagen membrane at day 10 (**A**–**D**) and day 30 (**E**–**H**) after implantation (all images: 400× magnification, scale bar = 20 µm).

The analysis of the immunohistochemically stained slides showed that more CD206-positive M1 macrophages were observable at this early study time point, compared to M2 positively stained with CD163 within the implantation beds of both materials (Figure 2A–D). Interestingly, no differences in the cell numbers of the CD163 nor the CD206 fractions have been microscopically observed between both study groups.

At day 30 post-implantation, the width of the reactive tissue wall adherent to the dPTFE membranes was clearly decreased (Figure 1C). Histopathological analyses showed that the reactive tissue was still composed of the same cell types, i.e., macrophages, granulocytes, and fibroblasts (Figure 1C). However, the numbers of granulocytes and macrophages have visibly been declined indicating a reduction of the degree of inflammation described at day 10 post implantation. At this time point, some single BMGCS have been found to be adherent to the dPTFE membranes. Still, no cell penetration or tissue ingrowth into the membranes was observed.

In case of the collagen membranes a similar tissue reaction compared to that observed at day 10 post-implantation has been detected (Figure 1D). Thus, macrophages, eosinophilic granulocytes and fibroblasts were found within the small walls of reactive tissue that are adherent to the material surfaces (Figure 1D). Moreover, the same cell types have microscopically been observed within the material bodies, but with significantly lower numbers compared to the surface-adherent connective tissue (Figure 1D). Only very low numbers of BMGCs sporadically found at the material surfaces have been found within the implantation beds of the collagen membrane. No ingrowth of complex tissue or any signs of a material breakdown have been detected.

The analysis of the immunohistochemically stained slides showed that the number of CD163-positive M2 macrophages has clearly decreased in the implantation beds of both biomaterials, while still no differences of the cell numbers in both groups could microscopically be detected (Figure 2E–H). Furthermore, it was observed that the numbers of CD206-positive M1 macrophages seemed to be comparable to the numbers of M2 macrophages in both groups (Figure 2E–H).

### 2.2. Histomorphometrical (Quantitative) Analysis

**Figure 3:**
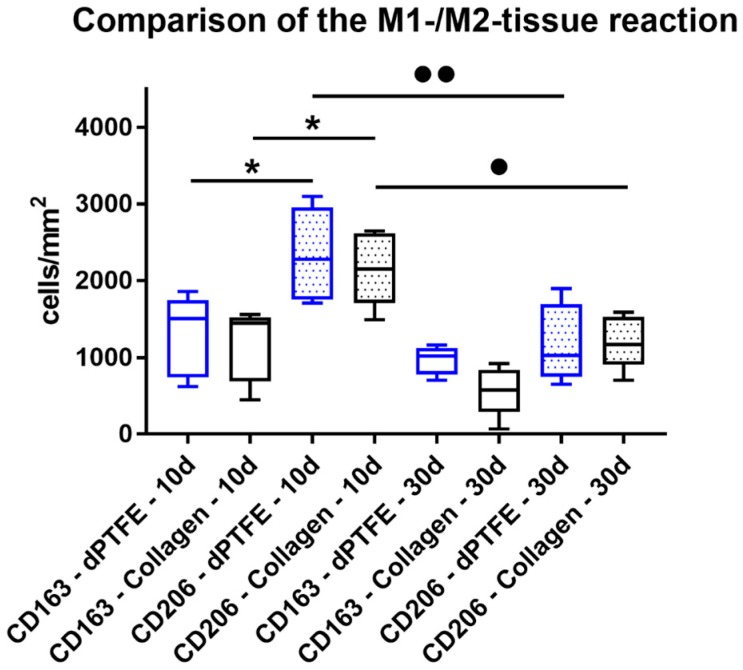
The results of the histomorphometrical analysis of the M1 and M2´macrophages within the implantation beds f both materials (*/● *p* < 0.05 and ●● *p* < 0.01).

The histomorphometrical analysis of the occurrence of pro- and anti-inflammatory cells showed that comparable numbers of CD163-positive M2 macrophages were detected in the implantation beds of the dPTFE membrane (1295.0 ± 529.8 cells/mm^2^) and the collagen membrane (1174.0 ± 476.9 cells/mm^2^) at day 10 after implantation (Figure 3). Furthermore, comparable numbers of CD206-positive M1 macrophages were found in the implantation beds of both biomaterials (dPTFE membrane: 2339.0 ± 608.6 cells/mm^2^; collagen membrane: 2159.0 ± 478.8 cells/mm^2^) at this study time point (Figure 3). In the implantation beds of both materials, significantly higher numbers of M1 macrophages (* *p* < 0.05) compared to the numbers of M2 macrophages per mm^2^ were detected at this time point (Figure 3).

At day 30 post implantation, comparable numbers of M2 macrophages have been found in the implantation beds of both membranes (dPTFE membrane: 968.0 ± 185.0 cells/mm^2^; collagen membrane: 568.2 ± 320.8 cells/mm^2^), but without any significance compared to the former study time point (Figure 3). Also, comparable numbers of M1 macrophages have been detected within the implant beds of both analyzed biomaterials (dPTFE membrane: 1182.0 ± 506.7 cells/mm^2^; collagen membrane: 1208.0 ± 346.5 cells/mm^2^) and no significant differences compared to the numbers of CD163-positive cells have been measured (Figure 3). Moreover, in case of both biomaterials, the numbers of M1 macrophages decreased significantly compared with day 10 after implantation (●● *p* < 0.001 and ● *p* < 0.05) (Figure 3).

## 3. Discussion

Different barrier membranes are available for guided bone regeneration (GBR) procedures, which can mainly be divided into resorbable und non-resorbable materials. Although resorbable membranes are preferred due to the avoidance of a second surgery, clinical situations such as bone defects outside the ridge contour, multi-walled bone defects, or vertical augmentations require maintenance of the spatial barrier, which can be achieved by the application of non-resorbable materials such as PTFE membranes [11]. Furthermore, ethical issues due to the xenogeneic origin of collagen membranes make PTFE a preferable GBR-membrane due to its synthetic origin [29]. PTFE-based membranes have been described in manifold to enable successful barrier functionality and associated successful bone regeneration, in various preclinical and clinical studies [1,30,31]. Moreover, the biocompatibility of PTFE materials has widely been studied, although limited knowledge about the underlying cellular responses exists. In this context, PTFE materials have been described to be bioinert [32,33]. However, it has been stated that no material implanted in living tissue is inert, because every biomaterial induces a tissue response [15]. Thus, the present study was conducted to examine the immune responses to a new dPTFE barrier membrane by the means of published histopathological and histomorphometrical analysis methods mainly focusing on immunohistochemical detection of M1- and M2-macrophages [24,25,26,27,28]. A collagen membrane described as a biocompatible and resorbable biomaterial was used as control [21,22,23].

The results of the present study show that the dPTFE membrane induced a tissue response, including inflammatory cell types such as macrophages and granulocytes, up to day 30 post implantation. Interestingly, the histomorphometrical detection of both macrophage subtypes showed that more CD206-positive M1 macrophages were present at day 10 after implantation, compared to macrophages expressing the M2 phenotype, and that this tissue reaction pattern was found to be comparable to the control collagen membrane. However, this early pro-inflammatory tissue reaction was not unexpected, as it is known that day 10 displays an early post-implantation phase, which still includes the reactions to the implantation procedure per se. More interestingly, the analysis showed a decrease of pro-inflammation reflected by the significant reduction of M1 macrophages at 30 days in both groups. Although no differences between the numbers of CD206-positive cells within the implantation beds of both biomaterials have been measured, the decrease of M1 macrophages was more pronounced in the case of the dPTFE membrane, as expressed by the higher significance level, as in case of the collagen membrane. Taken together, the significantly higher pro-inflammatory tissue response at day 10 after implantation was reduced at day 30 to a comparable level of M1 and M2 macrophages, even in the case of the dPTFE membrane.

In this context, it has been reported that an initial response of M1 macrophages that have been shown to lead to high levels of pro-inflammatory cytokine expressions to a biomaterial is a necessary process, while a prolonged proinflammatory response is associated with material failures, as it will induce a severe foreign body reaction or fibrous encapsulation [34]. In contrast, M2 macrophages consistently express anti-inflammatory cytokines that lead to a suppression of an inflammatory immune response, and that guide the tissue remodeling process [34]. These results lead to the conclusion that the dPTFE membrane altogether did induce an inflammatory tissue response that was comparable to the collagen membrane, which is considered to be biocompatible [35,36,37]. The tissue reaction to a non-resorbable biomaterial is comparable to that of a resorbable material, and this might be explained by the degradation mechanism of collagen-based biomaterials, which are mainly processed by physiological enzymes, such as matrix metalloproteinases (collagenases) [37]. This suggests that also in case of collagen membranes not a high level of inflammation is required for their degradation and, thus, the severity of pro- and anti-inflammation is comparable in the case of both biomaterials.

Furthermore, the question arises as to what may be the reasons for the found level of inflammation in the case of a non-resorbable biomaterial such as the analyzed dPTFE membrane. In this context, it has to be recalled that in healthy conditions, a physiological level of inflammation has also been shown to be present. Although the number of material-associated inflammatory cells is higher compared to the cell distribution in the surrounding connective tissue, it is questionable whether the level of expression of pro- and/or anti-inflammatory molecules is really increased in comparison to the immune cells of the healthy connective tissue. In this context, it has to be mentioned that the results of the present study can only give limited information about the degree of the inflammatory response as the immunohistochemical examination method does not allow for any assertion about the (level of) expression of the different cytokines or mediators by macrophages that are involved in the inflammatory tissue response to both analyzed biomaterials. Thus, the immunohistochemical detection method is not an analysis method that allows for the precise quantification of the severity of the foreign body reaction to the biomaterials, although it is a first indicator that allows an insight into the general tissue response to a biomaterial. This leads to the conclusion that a standardized in vitro test system including the cell types that are involved in the foreign body reaction to a biomaterial might also be necessary for analyzing biocompatibility, to prevent the rollout of inadequate biomaterials. Furthermore, specialized in vivo analysis methods, such as laser-assisted cell microdissection, which allows for the measurement of cytokine release from single cells or cell types, are important tools for biomaterial research and development [38,39].

Moreover, the time span up to day 30 post implantation might be not sufficient to make a final statement about the overall tissue reaction, which has to be regarded as a dynamic process. This means that the inflammatory cell or tissue response to the dPTFE membrane might ease after 30 days. However, the application of non-resorbable PTFE membranes is most often restricted to only 30 days after their application, which makes this investigation period justifiable [12,40].

Altogether, the results of the present study showed that the tissue response to the dPTFE membrane involves inflammatory macrophages. However, comparable cell numbers were found in the implant beds of a well-described collagen membrane, whose biocompatibility has been investigated and confirmed in different studies. Although these data indicate that the analyzed dPTFE membrane is not fully bioinert, they show that the device is biocompatible and thus may optimally support bone healing within the context of guided bone regeneration.

## 4. Materials and Methods

### 4.1. Barrier Membranes

#### 4.1.1. dPTFE Membrane (Permamem^®^)

**Figure 4:**
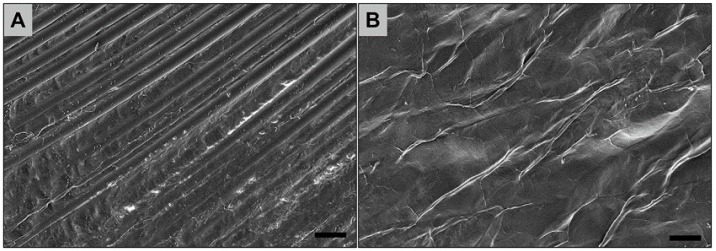
Exemplary scanning electron microscopy (SEM) images of the analyzed membranes. (**A**) dPTFE membrane (500× magnification, scale bar = 20 µm); (**B**) collagen membrane (500× magnification, scale bar = 20 µm).

The analyzed synthetic barrier membrane (permamem®, botiss biomaterials, Zossen, Germany) is made of non-resorbable high-density polytetrafluoroethylene (dPTFE) (Figure 4A). The membrane maintains its structural integrity during implantation and acts as an efficient barrier against bacterial and cellular penetration due to its small pore size [41]. The membrane fulfills the requirements of biocompatibility according to EN ISO 10993-1 and EN ISO 7405 [41].

#### 4.1.2. Pericardium-Based Collagen Membrane (Jason^®^ membrane)

The collagen membrane analyzed in the present study is based on native collagen originating from porcine pericardium (Jason^®^ membrane, botiss biomaterials, Zossen, Germany). The standardized manufacturing process includes an initial selection of the donor animals based on veterinary-controls. During the purification process, the pericardium undergoes a wet-chemical treatment, lyophilization, and sterilization by ethylene oxide gas. The collagen membrane exhibits a natural, multilayered structure with an increased content of collagen type III (Figure 4B). Also, this membrane has shown to fulfill the requirements of biocompatibility, according to EN ISO 10993-1 and EN ISO 7405 [41].

### 4.2. Scanning Electron Microscopy (SEM)

The (ultra-) structure of both biomaterials imaged by scanning electron microscopy (SEM) using a XL30 CP SEM (Philips, Amsterdam, The Netherlands).

### 4.3. In Vivo Study Design, Subcutaneous Implantation, and Explantation Procedure

The in vivo experiments and animal housing were conducted at the Faculty of Medicine (University of Niš, Serbia). The Local Ethical Committee (Faculty of Medicine, University of Niš, Serbia) authorized the described in vivo experiments, on the basis of the Veterinary Directorate of the Ministry of Agriculture, Forestry and Water Management of the Republic of Serbia issued the decision number 323-07-00278/2017-05/6 (Date: 13 July 2017). The animals were kept under standard conditions (water ad libitum, artificial light, and regular rat pellet) and standard pre- and postoperative care was ensured. The Local Ethical Committee (Faculty of Medicine, University of Niš, Serbia) authorized the described in vivo experiments.

In total, 20 female, 6–8 week-old BALB/c mice obtained from the Military Medical Academy (Belgrade, Serbia) were randomly allocated into two study groups. Each of the two study groups contained 10 experimental animals and five animals were used for implantation of the respective biomaterial per time point (*n* = 5), i.e., 10 and 30 days. The implantation was conducted following the protocol described by Barbeck et al. [24,25,26,28,42]. In brief, the animals were anesthetized via an intraperitoneal injection (10 mL ketamine (50 mg/mL) with 1.6 mL Xylazine (2%)). After shaving and disinfection, an incision down to the subcutaneous tissue within the rostral subscapular region was made. Subsequently, a subcutaneous pocket was bluntly built by scissors, and the biomaterials were implanted into the pocket. Afterwards, the wounds were sutured.

After the respective study time points, i.e., 10 and 30 days, the animals were euthanized with an overdose of the above-mentioned anesthetics and the implantation area, together with the surrounding tissue, were explanted. Subsequently, the explanted tissue was fixed using a 4% formalin solution for 24 h, and then placed into PBS for the following histological workup process.

#### 4.3.1. Histology and Immunohistochemistry

For the histological workup, the tissue explants were initially cut into two segments of identical dimensions and dehydrated using a series of increasing alcohol concentrations. After a xylol exposure, paraffin embedding was performed, followed by the preparation of sections with a thickness of 3–5 μm, which were prepared by means of a rotation microtome (SLEE, Mainz, Germany). Three sections of every tissue explant were used for histochemical stainings, i.e., haematoxylin and eosin (H&E), and Movat pentachrome and Alcian blue.

Furthermore, four additional sections of every tissue explants were used for the immunohistochemical detection of macrophages and their M1- and M2-subforms by means of antibodies against the pro- and anti-inflammatory molecules, i.e., hemoglobin scavenger receptor (CD163) and mannose receptor (MR, also known as CD206), based on previously published methods [28,42,43,44]. Briefly, the slides were initially treated with citrate buffer and proteinase K at pH8 for 20 min in a water bath at 96 °C, followed by equilibration using TBS-T buffer. Subsequently, the slides were prepared by H_2_O_2_ and avidin and biotin blocking solutions (Avidin/Biotin Blocking Kit, Vector Laboratories, Burlingame, CA, USA). Incubated with the respective first antibody for 30 min was conducted, followed by incubation with the secondary antibody (goat anti- rabbit IgG-B, sc-2040, 1:200, Santa Cruz Biotechnology, Shandon, CA, USA). Afterwards, the avidin–biotin–peroxidase complex (ThermoFisher Scientific, Dreeich, Germany) (30 min) was applied, and counterstaining by hematoxylin and blueing was conducted.

#### 4.3.2. Histological Analysis

The histological analyses to study the tissue–biomaterial interactions within the implantation beds of the biomaterials and their surrounding tissue were conducted using an Axio.Scope.A1 microscope (Zeiss, Oberkochen, Germany), as previously described [24,25,26,27,28]. These analyses focused on the evaluation of the following parameters within the framework of the early and the late tissue response related to the implants: fibrosis, hemorrhage, necrosis, vascularization, and the presence of neutrophils, lymphocytes, plasma cells, macrophages, and biomaterial-associated multinucleated giant cells (BMGCs). Finally, microphotographs were taken with an Axiocam 305 color connected to a computer system running the ZEN Core (Zeiss, Oberkochen, Germany) connected to the microscope.

#### 4.3.3. Histomorphometrical Analysis

The histomorphometrical analyses included the comparative measurements of the occurrence of anti-inflammatory and pro-inflammatory cells within the implant beds of the membranes, as previously described [24,25,26,27,28]. Briefly, “total scans” were generated with the aid of a specialized scanning microscope, which consists of an Axio Scope.A1 microscope combined with a Axiocam 305 color digital camera and an automatic scanning table (Maerzhaeuser, Wetzlar, Germany) connected to a computer system running the ZEN Core software (all: Zeiss, Oberkochen, Germany) containing the complete implant area, as well as the peri-implant tissue. The slides stained by the aforementioned immunohistochemical methods were digitized. To measure the extents of the cells, the amounts of these cells were manually counted using the “count tool” of the Zen Core software, and related to the total implant area (cells/mm^2^).

#### 4.3.4. Statistical Analyses

Quantitative data were shown as mean ± standard deviation after an analysis of variance (ANOVA), which enabled comparison of the data from the study groups via the GraphPad Prism 7.0d software (GraphPad Software Inc., La Jolla, California, USA). Statistical differences were designated as significant if the p-values were less than 0.05 (* *p* ≤ 0.05), and highly significant if the p-values were less than 0.01 (** *p* ≤ 0.01) or less than 0.001 (*** *p* ≤ 0.001).
